# Is a threshold-based model a superior method to the relative percent concept for establishing individual exercise intensity? a randomized controlled trial

**DOI:** 10.1186/s13102-015-0011-z

**Published:** 2015-07-04

**Authors:** Ali E. Wolpern, Dara J. Burgos, Jeffrey M. Janot, Lance C. Dalleck

**Affiliations:** 1 Recreation, Exercise, and Sport Science Department, Western State Colorado University, 600 N. Adams St., Gunnison, CO 81230 USA; 2 Department of Kinesiology, University of Wisconsin – Eau Claire, 105 Garfield Ave, PO Box 4004, Eau Claire, WI 54702 USA

**Keywords:** Cardiorespiratory fitness, Cardiovascular Disease, Exercise prescription, Primary prevention, VO_2_max

## Abstract

**Background:**

Exercise intensity is arguably the most critical component of the exercise prescription model. It has been suggested that a threshold based model for establishing exercise intensity might better identify the lowest effective training stimulus for all individuals with varying fitness levels; however, experimental evidence is lacking. The purpose of this study was to compare the effectiveness of two exercise training programs for improving cardiorespiratory fitness: threshold based model vs. relative percent concept (i.e., % heart rate reserve – HRR).

**Methods:**

Apparently healthy, but sedentary men and women (*n* = 42) were randomized to a non-exercise control group or one of two exercise training groups. Exercise training was performed 30 min/day on 5 days/week for 12weeks according to one of two exercise intensity regimens: 1) a relative percent method was used in which intensity was prescribed according to percentages of heart rate reserve (HRR group), or 2) a threshold based method (ACE-3ZM) was used in which intensity was prescribed according to the first ventilatory threshold (VT1) and second ventilatory threshold (VT2).

**Results:**

Thirty-six men and women completed the study. After 12weeks, VO_2_max increased significantly (*p* < 0.05 vs. controls) in both HRR (1.76 ± 1.93 mL/kg/min) and ACE-3ZM (3.93 ± 0.96 mL/kg/min) groups. Repeated measures ANOVA identified a significant interaction between exercise intensity method and change in VO_2_max values (*F* = 9.06, *p* < 0.05) indicating that VO_2_max responded differently to the method of exercise intensity prescription. In the HRR group 41.7 % (5/12) of individuals experienced a favorable change in relative VO_2_max (Δ > 5.9 %) and were categorized as responders. Alternatively, exercise training in the ACE-3ZM group elicited a positive improvement in relative VO_2_max (Δ > 5.9 %) in 100 % (12/12) of the individuals.

**Conclusions:**

A threshold based exercise intensity prescription: 1). elicited significantly (*p* < 0.05) greater improvements in VO_2_max, and 2). attenuated the individual variation in VO_2_max training responses when compared to relative percent exercise training. These novel findings are encouraging and provide important preliminary data for the design of individualized exercise prescriptions that will enhance training efficacy and limit training unresponsiveness.

**Trial registration:**

ClinicalTrials.gov Identifier: ID NCT02351713 Registered 30 January 2015.

## Background

Cardiorespiratory fitness, typically determined by maximal oxygen uptake (VO_2_max), is a fundamental measurement for the exercise physiologist and other health professionals. The magnitude of an individual’s cardiorespiratory fitness has been viewed as representative of overall health and studies have consistently demonstrated an inverse relationship between VO_2_max values and risk of cardiovascular disease and all-cause mortality [1, 2]. The “F.I.T.T.” principle is an acronym for the four components for exercise prescription: frequency, intensity, time (length), and type of exercise. Exercise intensity is arguably the most critical component of the exercise prescription model. Failure to meet minimal threshold values may result in lack of a training effect, while too high of an exercise intensity could lead to over-training and negatively impact adherence to an exercise program. The traditional reference standard for prescribing exercise intensity is expressed in terms of percentages of heart rate reserve (%HRR) or oxygen uptake reserve (%VO_2_R). This is considered the ‘*relative percent method*’. The American College of Sports Medicine (ACSM) currently recommends an exercise intensity of 40-59 % HRR/VO_2_R for improving and maintaining cardiorespiratory fitness [3].

Nevertheless, despite the large base of Category A evidence [4] supporting the ACSM relative percent concept recommendation for prescribing exercise intensity, there is concern that the relative percent concept approach consists of a very large range of acceptable percentages [5] and also fails to take into account individual metabolic responses (e.g., blood lactate) to exercises [6]. For example, it has been demonstrated [7–9] that there is considerable individual variation in the blood lactate response to exercise when intensity is anchored to relative percent concepts (e.g., %HRR, %VO_2_max). In turn, it has been suggested that this heterogeneous variation in the metabolic strain of each exercise session may ultimately yield individual variation in training adaptations; thus resulting in positive responders and non-responders to chronic exercise training [10].

Alternatively, it has been suggested that a ‘*threshold based model*’ for establishing exercise intensity might better identify the lowest effective training stimulus and elicit comparable relative metabolic strain across individuals with varying fitness levels [5, 6, 9]. Indeed, the American Council on Exercise (ACE) [11] recommends a ‘*threshold based model*’ approach to prescribing exercise intensity in its *ACE three-zone training model*. However, it has been acknowledged elsewhere that the experimental evidence supporting a ‘*threshold based model*’ approach to exercise training is lacking [11]. Moreover, it remains to be determined if a threshold based training model will attenuate individual variation in training responses when compared to exercise training prescription anchored to relative percent methods [10].

Therefore, the purpose of this study was to compare the effectiveness of two exercise training programs for improving cardiorespiratory fitness: a threshold based training model vs. the relative percent method (i.e., %HRR).

It was hypothesized that:

1. The threshold based training model would elicit greater mean changes in cardiorespiratory fitness (as measured by VO_2_max) when compared to the relative percent method.
2. Participants in the threshold based training model group would be more likely to have favorable VO_2_max responses; while comparatively, participants in the relative percent method group would be more likely to experience a VO_2_max nonresponse to exercise training.

## Methods

### Participants

Forty-two nonsmoking men and women (18 to 54 years) were recruited from the student and faculty population of a local university, as well as the surrounding community, via advertisement through the university website, local community newspaper, and word-of-mouth. Participants were eligible for inclusion into the study if they were low-to-moderate risk as defined by the ACSM and sedentary. Participants were considered sedentary if they reported not participating in at least 30 min of moderate intensity physical activity on at least three days of the week for at least three months [3]. Participants were also eligible for inclusion into the study if they verbally agreed to continue previous dietary habits and not perform additional exercise beyond that required for the present study. Exclusionary criteria included evidence of cardiovascular pulmonary, and/or metabolic disease as determined by medical history questionnaire. This study was approved by the Human Research Committee at Western State Colorado University. Each participant signed an informed consent form prior to participation.

### Baseline, midpoint, and post-program experimental testing procedures

Measurements of all primary and secondary outcome variables were obtained both before and after the exercise training intervention. Additionally, a measure of the primary outcome variable (maximal oxygen uptake – VO_2_max) was also obtained at midpoint. Secondary outcome variables consisted of resting heart rate and blood pressure, and basic anthropometric measures including height, weight, waist circumference, and skinfolds. Fasting blood lipid and blood glucose measurements were also performed. All measurements were obtained by following standardized procedures as outlined elsewhere [3]. Procedures for each measurement are also briefly described below. Prior to testing participants refrained from all food and drink other than water for 12 h. Participants were also instructed to refrain from strenuous exertion 12 h prior to testing. All post-program testing took place within 1 to 4 days of the last exercise training session.

#### Resting heart rate and blood pressure measurement

The procedures for assessment of resting heart rate and blood pressure outlined elsewhere were followed [3]. Briefly, participants were seated quietly for 5 min in a chair with a back support with feet on the floor and arm supported at heart level. Resting heart rate was obtained via manual palpation of radial artery in the left wrist and recording the number of beats for 60 s. The left arm brachial artery blood pressure was measured using a sphygmomanometer in duplicate and separated by 1-min. The mean of the two measurements was reported for baseline and post-program values.

#### Anthropometric measurements

Participants were weighed to the nearest 0.1 kg on a medical grade scale and measured for height to the nearest 0.5 cm using a stadiometer. Percent body fat was determined via skinfolds [3]. Skinfold thickness was measured to the nearest ± 0.5 mm using a Lange caliper (Cambridge Scientific Industries, Columbia, MD). All measurements were taken on the right side of the body using standardized anatomical sites (three-site) for men and women. These measurements were performed until two were within 10 % of each other. Waist circumference measurements were obtained using a cloth tape measure with a spring loaded-handle (Creative Health Products, Ann Arbor, MI). A horizontal measurement was taken at the narrowest point of the torso (below the xiphoid process and above the umbilicus). These measurements were taken until two were within 0.5 mm of each other.

#### Fasting blood lipid and blood glucose measurement

All fasting lipid and blood glucose analyses were collected in duplicate and performed at room temperature. The mean of the two measurements was reported for baseline and post-program values. Participants’ hands were washed with soap and rinsed thoroughly with water, then cleaned with alcohol swabs and allowed to dry. Skin was punctured using lancets and a fingerstick sample was collected into heparin-coated 40 μl capillary tube. Blood was allowed to flow freely from the fingerstick into the capillary tube without milking of the finger. Samples were then dispensed immediately onto commercially available test cassettes for analysis in a Cholestech LDX System (Alere Inc., Waltham, MA) according to strict standardized operating procedures. The LDX Cholestech measured total cholesterol, high density lipoprotein (HDL) cholesterol, low density lipoprotein (LDL) cholesterol, triglycerides, and blood glucose in fingerstick blood. A daily optics check was performed on the LDX Cholestech analyzer used for the study. Independent studies have provided data to indicate that the Cholestech LDX system has excellent reproducibility with standard clinical laboratory measurement of plasma lipids and lipoproteins [12, 13] and meets the National Cholesterol Education Program Adult Treatment Panel III (NCEP-ATP) criteria for accuracy and reproducibility [14].

#### Maximal exercise testing

Participants completed a modified-Balke, pseudo-ramp graded exercise test (GXT) on a power treadmill (Powerjog GX200, Maine). Participants walked or jogged at a self-selected pace. Treadmill incline was increased by 1 % every minute until the participant reached volitional fatigue. Participant HR was continuously recorded during the GXT via a chest strap and radio-telemetric receiver (Polar Electro, Woodbury, NY, USA). Expired air and gas exchange data were recorded continuously during the GXT using a metabolic analyzer (Parvo Medics TrueOne 2.0, Salt Lake City, UT, USA). Before each exercise test, the metabolic analyzer was calibrated with gases of known concentrations (14.01 ± 0.07 % O_2_, 6.00 ± 0.03 % CO_2_) and with room air (20.93%O_2_ and 0.03 % CO_2_) as per the instruction manual. Volume calibration of the pneumotachometer was done via a 3-Litre calibration syringe system (Hans-Rudolph, Kansas City, MO, USA). The last 15 s of the GXT were averaged – this was considered the final data point. The closest neighbouring data point was calculated by averaging the data collected 15 s immediately before the last 15 s of the test. The mean of the two processed data points represented VO_2_max. Maximal HR was considered to be the highest recorded HR in beats per minute (bpm) during the GXT. Participant heart rate reserve (HRR) was determined by taking the difference between maximal HR and resting HR.

#### Determination of ventilatory thresholds

Determination of both the first ventilatory threshold (VT1) and second ventilatory threshold (VT2) were made by visual inspection of graphs of time plotted against each relevant respiratory variable (according to 15 s time-averaging). The criteria for VT1 was an increase in VE/VO_2_ with no concurrent increase in VE/VCO_2_ and departure from the linearity of VE. The criteria for VT2 was a simultaneous increase in both VE/VO_2_ and VE/VCO_2_ [15]. All assessments were done by two experienced exercise physiologists. In the event of conflicting results, the original assessments were reevaluated and collectively a consensus was agreed upon.

### Randomization and exercise intervention

**Fig. 1:**
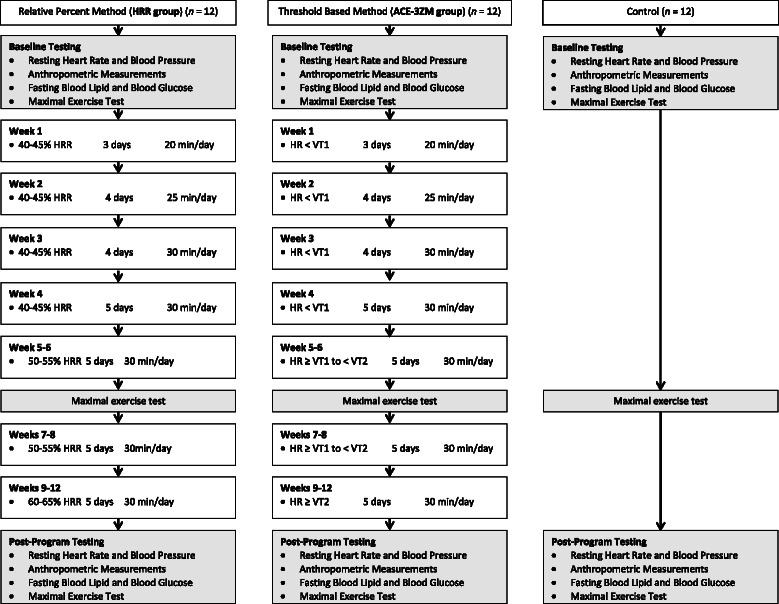
Flow chart of experimental procedures and exercise prescription for each of the two exercise training groups. HR, heart rate; HRR, heart rate reserve; VT1, first ventilatory threshold; VT2, second ventilatory threshold

After the completion of baseline testing, participants were randomized to a non-exercise control group or one of two exercise training groups according to a computer generated sequence of random numbers that was stratified by sex (Fig. 1). This was a double-blind research design in that participants were unaware of the group to which they had been assigned. Likewise, the researchers specifically responsible for testing and supervision of exercise sessions were unaware of the group to which participants had been allocated. Participants randomized to the exercise training groups performed 12weeks of exercise training according to one of two exercise intensity regimens: 1) a relative percent method was used in which intensity was prescribed according to percentages of HRR (HRR group), or 2) a threshold-based method (ACE-3ZM) was used in which intensity was prescribed according to VT1 and VT2 as recommended by ACE in its three-zone model [11]. The exercise prescription details for each training group over the course of the 12weeks training period is presented in Fig. 1.

Each group performed a similar frequency and duration of exercise training. All exercise training sessions for each treatment group were performed on a treadmill. Overall, the exercise prescription was intended to fulfill the consensus recommendation of 150 min/week [4]. The exercise prescription for exercise intensity method differed between treatment groups. The HRR group was prescribed exercise intensity according to a percentage of HRR. Conversely, the ACE-3ZM group was prescribed exercise intensity according to ventilatory threshold. In both exercise training groups a target heart rate (HR) coinciding with either the prescribed HRR or prescribed VT (Fig. 1) was used to establish a specific exercise training intensity for each exercise session. In the ACE-3ZM group target HR for each training zone (Fig. 1) was established in the following manner:

- **Wk 1–4 (HR < VT1):** target HR = HR range of 10–15 bpm just below VT1
- **Wk 5–8 (HR ≥ VT1 to < VT2):** target HR = HR range of 10–20 bpm above VT1 and below VT2
- **Wk 9–12 (HR ≥ VT2):** target HR = HR range of 10–15 bpm just above VT2

Exercise training was progressed according to recommendations made elsewhere by the ACSM [3] and ACE [11]. Polar HR monitors (Polar Electro Inc., Woodbury, NY) were used to monitor HR during all exercise sessions. Researchers adjusted treadmill workload accordingly during each exercise session to ensure actual HR responses aligned with target HR.

### Statistical analyses

All analyses were performed using SPSS Version 22.0 (Chicago, IL) and GraphPad Prism 6.0. (San Diego, CA). Sample size was projected with change in VO_2_max as the main outcome variable. The means and standard deviation of a previous study [16] were examined and the effect size of this study was calculated. Assuming that a power of 0.90 was needed and the calculated effect size for change in VO_2_max was 0.8, it was determined that approximately 12 subjects would be needed for each of the three groups [17]. Further, we assumed there would be an approximate 20 % dropout rate based on findings from one of our previous exercise training studies [18]. Accordingly, we recruited and randomized an additional three participants to each of the exercise training groups to account for potential attrition.

Measures of centrality and spread are presented as mean ± standard deviation (SD). All baseline-dependent variables were compared using general linear model (GLM) ANOVA and, where appropriate, Tukey post hoc tests. Within-group comparisons were made using paired *t*-tests. Because baseline, 6weeks, and post-program data were available, the effect of exercise training on cardiorespiratory fitness (VO_2_max) was determined using repeated-measures GLM-ANOVA with exercise intensity method (HRR or ACE-3ZM) as the between-subjects factor. All other between-group 12weeks changes were analyzed using GLM-ANOVA and, where appropriate, Tukey post hoc tests. The assumption of normality was tested by examining normal plots of the residuals in ANOVA models. Residuals were regarded as normally distributed if Shapiro-Wilk tests were not significant [17].

Delta values (Δ) were calculated (post-program minus baseline value divided by baseline value) for percent change in relative VO_2_max (%) and participants were categorized as: ‘1’ = responders (% Δ > 5.9 %) or ‘0’ = non-responders (Δ ≤ 5.9 %) to exercise training using a day-to-day variability, within subject coefficient of variation (CV) criterion applied previously in the literature [6, 19]. Chi-square (*χ*^2^) tests were subsequently used to analyze the point prevalence of responders and non-responders to exercise training separated by exercise intensity group (i.e., HRR and ACE-3ZM) between baseline and post-program. The probability of making a Type I error was set at *p* ≤ 0.05 for all statistical analyses.

## Results

All analyses and data presented in the results are for those participants who completed the investigation. At baseline, treatment (HRR and ACE-3ZM) and non-exercise control groups did not differ significantly in physical or physiological characteristics. The physical and physiological characteristics for participants are shown in Table 1.

**Table 1:** Physical and physiological characteristics at baseline and 12weeks for control, HRR, and ACE-3ZM groups. (Values are mean ± SD)

Parameter	Control group		HRR group		ACE-3ZM group	
	(*n* = 12; women = 7, men = 5)		(*n* = 12; women = 6, men = 6)		(*n* = 12; women = 6, men = 6)	
	Baseline	12weeks	Baseline	12weeks	Baseline	12weeks
Age (yr)	33.5 ± 7.0	____	33.0 ± 9.8	____	31.7 ± 9.6	____
Height (cm)	165.5 ± 9.6	____	170.3 ± 7.1	____	169.3 ± 12.7	____
Body mass (kg)	72.1 ± 9.6	72.2 ± 9.2	73.3 ± 12.4	73.8 ± 12.2*	72.8 ± 15.7	72.8 ± 15.5
Waist circumference (cm)	81.8 ± 8.4	82.0 ± 8.2	81.7 ± 8.3	82.5 ± 8.4*	80.1 ± 11.3	79.8 ± 10.8
Body fat (%)	19.8 ± 5.9	20.6 ± 5.4*	16.1 ± 5.1	16.6 ± 5.2*	19.5 ± 6.4	18.7 ± 6.2*‡
Resting HR (b⋅min^−1^)	64.6 ± 12.2	65.2 ± 8.9	59.4 ± 11.6	59.3 ± 7.6	69.1 ± 7.4	65.1 ± 3.7
Maximal HR (b⋅min^−1^)	179.0 ± 14.0	180.6 ± 12.3	180.9 ± 14.3	182.3 ± 12.0	182.4 ± 12.8	184.5 ± 12.9*
VO_2_max (mL⋅kg^−1^⋅min^−1^)	30.4 ± 6.4	29.9 ± 6.0	34.9 ± 5.3	36.6 ± 5.4*†	34.3 ± 9.0	38.3 ± 9.2*‡
Systolic BP (mmHg)	118.4 ± 9.7	120.0 ± 8.9	117.4 ± 6.8	119.3 ± 5.3	117.9 ± 9.8	115.9 ± 7.1
Diastolic BP (mmHg)	78.4 ± 8.9	80.5 ± 4.9	79.4 ± 3.7	78.6 ± 4.8	73.7 ± 11.2	74.0 ± 9.1
Total cholesterol (mg⋅dL^−1^)	192.1 ± 32.9	194.4 ± 28.0	188.4 ± 23.8	188.9 ± 23.8	179.0 ± 47.1	168.3 ± 30.0
HDL cholesterol (mg⋅dL^−1^)	52.0 ± 21.9	50.5 ± 19.1	53.3 ± 14.5	55.0 ± 14.7	51.8 ± 20.9	60.2 ± 20.3*‡
LDL cholesterol (mg⋅dL^−1^)	111.0 ± 29.2	113.1 ± 25.8	110.2 ± 25.6	109.7 ± 22.4	92.2 ± 28.8	85.9 ± 27.7*
Triglycerides (mg⋅dL^−1^)	115.4 ± 41.5	123.2 ± 40.8	118.2 ± 70.4	120.3 ± 57.5	94.8 ± 45.9	97.5 ± 33.0
Blood Glucose (mg⋅dL^−1^)	88.5 ± 5.5	89.8 ± 7.1	90.1 ± 5.1	89.8 ± 5.1	92.4 ± 10.2	90.1 ± 4.9

*Within-group change is significantly different from baseline, *p* < 0.05; † Change from baseline is significantly different than control group, *p* < 0.05; ‡ Change from baseline is significantly different than control and HRR groups, *p* < 0.05

The exercise prescription in both treatment groups was well tolerated for the 24 of 30 participants who completed the study. Six participants were unable to complete the study for the following reasons: injury outside the study (*n* = 2), illness (*n* = 2), out-of-town move (*n* = 1), and personal reasons (n = 1). Dropout was similar in both treatment groups. Overall, there was excellent adherence to the total number of prescribed training sessions: HRR group – mean, 90.6 % (range, 76.8-100 %) and ACE-3ZM group – mean, 89.3 % (range, 78.6-100 %). Additionally, adherence to the prescribed exercise intensity for both treatment groups throughout the duration of the intervention was excellent (Table 2).

**Table 2:** Prescribed and actual exercise intensity for HRR and ACE-3ZM groups throughout the 12weeks exercise intervention

	HRR Group (*n* = 12)			ACE-3ZM Group (*n* = 12)		
Week	*Prescribed intensity*	*THR*	*Actual HR*	*Prescribed intensity*	*THR*	*Actual HR*
1	40-45 % HRR	109 ± 15 to 116 ± 15	113 ± 15	HR < VT1	126 ± 13 to 136 ± 13	131 ± 16
2	40-45 % HRR	109 ± 15 to 116 ± 15	115 ± 13	HR < VT1	126 ± 13 to 136 ± 13	133 ± 12
3	40-45 % HRR	109 ± 15 to 116 ± 15	113 ± 15	HR < VT1	126 ± 13 to 136 ± 13	131 ± 13
*4*	40-45 % HRR	109 ± 15 to 116 ± 15	112 ± 13	HR < VT1	126 ± 13 to 136 ± 13	135 ± 12
*5-6*	*50*-55 % HRR	116 ± 14 to 121 ± 15	118 ± 14	HR ≥ VT1 to < VT2	137 ± 12 to 147 ± 15	141 ± 9
*7-8*	*50*-55 % HRR	117 ± 16 to 125 ± 16	123 ± 16	HR ≥ VT1 to < VT2	140 ± 14 to 151 ± 13	145 ± 14
9-12	*60*-65 % HRR	127 ± 16 to 133 ± 16	132 ± 16	HR ≥ VT2	153 ± 9 to 161 ± 10	155 ± 11

Values are mean ± SD. *HR* heart rate, *HRR* heart rate reserve, *THR* target heart rate, *VT1* first ventilatory threshold, *VT2* second ventilatory threshold

**Fig. 2:**
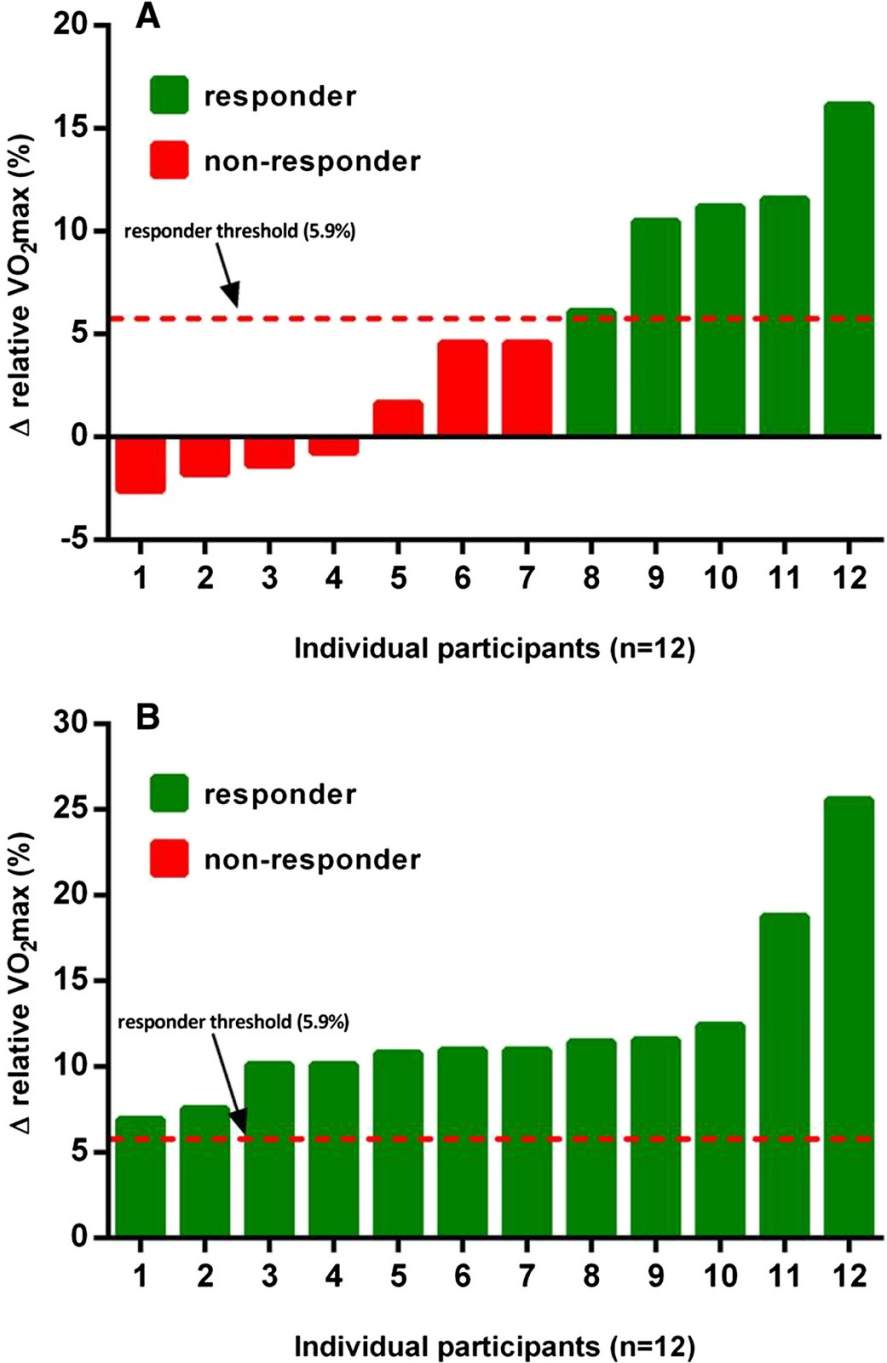
Individual variability in relative VO_2_max response (% change) to exercise training in the HRR (A) and ACE-3ZM (B) groups

After 12 week, changes in body mass, waist circumference, resting HR, systolic and diastolic blood pressure, total cholesterol, LDL cholesterol, triglycerides, and blood glucose were not significantly different (*p* > 0.05) in either the HRR or ACE-3ZM groups when compared with the control group. In contrast, changes in VO_2_max from baseline to 12weeks in the HRR group were significantly greater (*p* < 0.05) when compared with the control group. Moreover, changes in body fat percentage, VO_2_max, and HDL cholesterol in the ACE-3ZM group were significantly more favorable (*p* < 0.05) when compared to both the HRR and control groups. After 12weeks, VO_2_max increased significantly (*p* < 0.05 vs. controls) in both HRR (1.76 ± 1.93 mL⋅kg^−1^⋅min^−1^) and ACE-3ZM (3.93 ± 0.96 mL⋅kg^−1^⋅min^−1^) groups. Repeated measures ANOVA identified a significant interaction between exercise intensity method and change in VO_2_max values (*F* = 9.06, *p* < 0.05) indicating that VO_2_max responded differently to the method of exercise intensity prescription (Fig. 2).

### Prevalence of VO_2_max non-responders and responders

The point prevalence of responders and non-responders to exercise training in both the HRR and ACE-3ZM groups are shown in Fig. 2. In the HRR group 41.7 % (5/12) of individuals experienced a favorable change in VO_2_max (Δ > 5.9 %) and were categorized as responders. Alternatively, 58.3 % (7/12) of individuals in the HRR group experienced an undesirable change in VO_2_max (Δ ≤ 5.9 %) and were categorized as non-responders to exercise training. There were no significant differences (p < 0.05) in several potential influencing factors of responder/non-responder, including age, baseline VO_2_max, exercise adherence, and sex. In the ACE-3ZM group the prevalence of individuals who experienced a favorable change in VO_2_max was significantly (p < 0.05) greater when compared to the HRR group. Indeed, exercise training in the ACE-3ZM group elicited a positive improvement in VO_2_max (Δ > 5.9 %) in 100 % (12/12) of the individuals.

## Discussion

The major findings from the present study were as follows: 1) threshold based exercise intensity prescription elicited significantly (*p* < 0.05) greater improvements in VO_2_max when compared to a relative percent exercise intensity prescription following 12weeks of exercise training, and 2) threshold based exercise training attenuated the individual variation in VO_2_max training responses when compared to relative percent exercise training as evidenced by the significantly reduced (*p* < 0.05) point prevalence of exercise training non-responders in the ACE-3ZM treatment group. Therefore, these current results support both our research hypotheses and underscores the importance of establishing exercise training intensity relative to threshold measurements. To our knowledge, this is the first prospective, randomized, controlled trial to compare individual variation in VO_2_max training responses following threshold-related training versus training at a %HRR [10].

### Heterogeneity in VO_2_ Response to Exercise Training

Although it is well accepted that regular exercise has positive effects on cardiorespiratory fitness and numerous other health outcomes related to cardiovascular morbidity and mortality [4], it has also been recently highlighted that considerable heterogeneity exists with respect to the magnitude of VO_2_ response to chronic exercise training [20]. Indeed, wide variability (−33.2 % to +58 %) in the individual VO_2_ response to exercise training has been previously described in the literature [18, 21–23]. It has been reported that age, sex, race, and initial VO_2_max do not influence the heterogeneity in the individual VO_2_ response to exercise training [21, 23]. Results from the present study corroborate these earlier findings as the potential influencing factors of age, baseline VO_2_max, exercise adherence, and sex were not significantly different between VO_2_max responders and non-responders. In contrast, it has been reported that genetics is responsible for 47 % of the change in VO_2_max [24]. Additionally, it has previously been demonstrated that one of the most important predictors of a positive VO_2_ response to exercise training is a greater volume of exercise [25].

Recently, it has been suggested that the method of exercise intensity prescription may underpin the inter-individual variation in VO_2_ response to exercise training [10]. Those previous studies [21–23, 25] that have reported wide variability in the individual VO_2_ response to exercise training have used one of several relative exercise intensity methods, including %HRmax, %HRR, or %VO_2_max. However, it has been demonstrated that these exercise intensity prescription methods elicit large inter-individual variation in the metabolic responses to exercise training [10, 26]. On this basis, it has been postulated that the individual variation in metabolic response will subsequently lead to differences in the overall homeostatic stress from each training session which will ultimately result in heterogeneity in the exercise training response (i.e., change in VO_2_max). Alternatively, it has been suggested that use of a threshold based method for establishing exercise intensity might better normalize the metabolic stimulus for individuals with varying fitness levels [5, 11]. Results from the present study provide experimental evidence to support the merits of a threshold based method for prescription of exercise intensity. Consistent with previous studies [6, 26], this current study found considerable heterogeneity in terms of VO_2_max responders (41.7 %) and non-responders (58.3 %) when exercise intensity was prescribed in relative terms as a %HRR. In contrast, we demonstrated a consistent positive VO_2_max response to exercise training (100 % responders) when a threshold based exercise intensity prescription method was employed. Taken together, the results of this novel study are encouraging and provide important preliminary data for the design of individualized exercise prescriptions that will enhance training efficacy and limit training unresponsiveness [27].

### Primary prevention of chronic disease perspective

In the past decade low cardiorespiratory fitness has garnered considerable attention as an independent and powerful predictor of CVD risk and premature mortality. For example, Williams [2] showed in a meta-analysis that there was a marked increase in relative risk for CVD in the lowest quartile of cardiorespiratory fitness. More recently Blair [28] estimated that low cardiorespiratory fitness accounted for more overall deaths when compared to deaths which could be attributed to traditional CVD risk factors, such as obesity, smoking, hypertension, high cholesterol, and diabetes. Accordingly, the ACE-3ZM change in VO_2_max results from the current study have novel clinical and public health relevance, as a large number of adults fall into clinically-defined low cardiorespiratory fitness categories and therefore demonstrate increased CVD risk [29]. Importantly, exercise training in the ACE-3ZM group elicited a positive improvement in VO_2_max in 100 % (12/12) of the individuals. Overall, VO_2_max was improved on average by 1.1 METs (range +0.65 to +1.63 METs) in the ACE-3ZM group following 12weeks of exercise training. These improvements likely have important long-term prevention implications as a recent study reported a 1 MET increase in VO_2_max was associated with an 18 % reduction in deaths due to CVD [30].

### Methodological considerations

There are several limitations to the present study that warrant further discussion. First, overall sample size in our study is lower than other major training studies in the literature [16, 25]. However, advantages of a smaller sample size were the ability to better supervise the exercise program and more closely interact with participants on a daily basis during exercise sessions [31]. In particular, the adherence to the prescribed exercise intensity and program was excellent for both exercise treatment groups (Table 2). Second, the present study (12weeks) was also relatively modest in duration compared to previous training investigations [16, 25, 31]. Nevertheless, the significant improvements in VO_2_max found in the present study indicate that a 12weeks exercise training period is a sufficient timeframe to demonstrate significant training effects. Future research is needed to confirm the possibility of additional improvements in VO_2_max following an exercise training program similar in characteristics to the present study but for prolonged durations. Third, while participants were instructed to maintain their regular dietary intake during the 12weeks intervention, diet intake was not strictly controlled for in this study. Moreover, physical activity outside of the training program was not monitored and thus may have influenced the current findings.

## Conclusion

There is a wealth of previous research demonstrating an independent, inverse, dose–response relationship between cardiorespiratory fitness and all-cause and CVD mortality [1, 3, 32, 33]. Moreover, cardiorespiratory fitness has been called the ultimate marker for risk stratification and health outcomes [34]. In the present study a threshold based exercise intensity prescription elicited significantly greater improvements in VO_2_max and attenuated the individual variation in VO_2_max training responses when compared to relative percent exercise training. These novel findings are encouraging and provide important preliminary data for the design of individualized exercise prescriptions that will enhance training efficacy and limit training unresponsiveness.
